# Elite Male Volleyball Players Are at Risk of Insufficient Energy and Carbohydrate Intake

**DOI:** 10.3390/nu13051435

**Published:** 2021-04-24

**Authors:** Erik Sesbreno, Christine E. Dziedzic, Jennifer Sygo, Denis P. Blondin, François Haman, Suzanne Leclerc, Anne-Sophie Brazeau, Margo Mountjoy

**Affiliations:** 1 l’Institut National du Sport du Québec, Montréal, QC H1V 3N7, Canada; sleclerc@insquebec.org; 2 French-Speaking Olympic Sports Medicine Research Network (ReFORM), Montréal, QC H1V 3N7, Canada; 3 School of Human Nutrition, McGill University, Montréal, QC H9X 3V9, Canada; anne-sophie.brazeau@mcgill.ca; 4 Buffalo Sabres, Buffalo, NY 14203, USA; cdziedzic@sabres.com; 5 Athletics Canada, Ottawa, ON K1G 6C9, Canada; jsygo@rogers.com; 6 Department of Medicine, Division of Neurology, Faculty of Medicine and Health Sciences, Centre de Recherche du CHUS, Université de Sherbrooke, Sherbrooke, QC J1H 5N4, Canada; denis.p.blondin@gmail.com; 7 School of Human Kinetics, University of Ottawa, Ottawa, ON K1N 6N5, Canada; fhaman@uottawa.ca; 8 Fédération Internationale de Natation (FINA), 1007 Lausanne, Switzerland; mmsportdoc@mcmaster.ca; 9 Department of Family Medicine, Michael G. DeGroote School of Medicine, McMaster University, Hamilton, ON L8S 4L8, Canada

**Keywords:** low energy availability, nutrition recommendations, elite athletes, RED-S, carbohydrate energy availability, eating behaviour

## Abstract

Elite volleyball athletes experience significant physical and psychological demands during the competitive season. The aim was to compare the dietary intake of male volleyball athletes with recommendations for sport and health, and to examine the association of physique traits and knee health on eating behaviours and of eating behaviours on reported dietary intake. Using a retrospective cross-sectional design, 22 male athletes from a national indoor volleyball program underwent anthropometric, dual-energy X-ray absorptiometry and resting metabolic rate testing, 4-day dietary intake and hematological analysis, and also completed the three-factor eating questionnaire–R18 for eating behaviours and the Victorian Institute of Sport Assessment—patellar tendon (VISA-P) questionnaire for knee health. Most players under-consumed energy compared to reference guidelines, secondary to under-consuming carbohydrate for exercise. The primary eating behaviour was cognitive restraint, which was associated with body mass index and ectomorphy. Emotional eating behaviour was associated with VISA-P. Differences in emotional and cognitive restraint eating behaviours did not impact dietary intake. The findings suggest that players are at risk of an impaired ability to adapt to and recover from training during an important segment of the competitive season. Future work should explore the presence of low energy availability in elite male volleyball players.

## 1. Introduction

Elite volleyball athletes experience significant physical and psychological demands during the competitive season. They participate in practices and matches that are 90+ min in duration, with frequent explosive movements, and the weekly expectations and commitments for training and competition during the in-season challenge their perception of wellness [1,2]. Consequently, these athletes require a robust nutritional program because mismanaging dietary intake may have undesirable outcomes on performance, physique, training responses and the prevention of, and return from, injury [3,4,5]. It is, therefore, important to examine the eating habits of elite volleyball players to optimise performance on the court.

The assessment of dietary intake is helpful to identify adherence to sport nutrition recommendations [6]. Elite female indoor volleyball players have been reported to consume inadequate daily energy and carbohydrate, whereas only energy intake was insufficient in male players [7,8,9,10]. However, few have compared results to contemporary energy and macronutrient intake guidelines for sport, and the paucity of information on male players limits generalised application. Not only is it important to assess the dietary intake of elite male players, but also to assess eating behaviours, as they may influence nutrient intake.

The three-factor eating questionnaire–R18 (TFEQ-R18) has been used to characterise cognitive restraint and emotional eating behaviours in both the general and athletic populations in order to understand interactions between eating and health risks [11,12]. The assessment of restrained eating would also be useful in jumping sports, given the potential negative implications of excessive body fat for jump performance [13]. Furthermore, the description of dietary intake associated with restrained eating behaviour would help to inform the risk assessment of poor performance and injuries associated with low energy availability (LEA), and macronutrient and/or micronutrient intake inadequacy [4,5,14,15,16]. Despite the importance of this assessment, it may be beneficial to concurrently examine eating behaviours associated with injury.

Patellar tendinopathy has been observed in many elite sports, affecting an estimated 45% of volleyball players [17]. Patellar tendinopathy may limit players from participating in exercise while ~50% of athletes with the injury are forced to quit their sport career [18]. A potential association between persistent pain and emotional eating may occur, and emotional eating has been associated with increased energy intake, body weight, and body fat in non-athletic populations [19]. The influence of pain on eating behaviour has rarely been evaluated in elite athletes, and given the potential consequences for weight and body composition, a preliminary evaluation is warranted.

Therefore, the present study aimed to achieve the following: 1. Compare reported dietary intake with nutrition recommendations for sport and health. 2. Determine the potential association of cognitive restraint on physique traits and emotional eating on knee health. 3. Assess the association of cognitive restraint and emotional eating behaviours on dietary intake in elite volleyball male athletes.

## 2. Materials and Methods

### 2.1. Recruitment

A total of 22 elite male players, 18 years of age or older, were recruited for the study. Athletes were identified as elite if they received Government sport funding for the 2019 season. These athletes competed in the Fédération Internationale de Volleyball World Championships or Olympic Games, were part of the senior national team program, or were part of the “NextGen” (Next Generation) program with the potential of qualifying for a roster position on the senior men’s national team. The exclusion criterion was athletes inactive to play due to injury.

### 2.2. Experimental Design

In a retrospective study design, data collected included demographics, responses from the TFEQ R-18, 4-day dietary intake record, blood work, measured resting metabolic rate (mRMR), dual-energy X-ray absorptiometry (DXA) scan, surface anthropometry and the Victorian Institute of Sport Assessment questionnaire—patellar tendon (VISA-P) over a 2-week observation period prior to the 2020 Olympics qualifiers in August 2019. Athletes were training approximately 20 h per week at the national team training centre. Athletes were provided with details on the nature of the study, and all participants provided written informed consent in accordance with the declaration of Helsinki. Ethics approval was obtained through the University of Ottawa research ethics committee (H-10-19-5054). 

### 2.3. Measured Resting Metabolic Rate

Resting metabolic rate was measured, in a fasting state, to operate the Goldberg cut-off to assess cases of over- or under-reporting as previously described [20]. Whole body mRMR and substrate utilisation were determined from the rates of oxygen consumption (VO_2_) and carbon dioxide production (VCO_2_), measured using a flow-through open circuit respirometry system with a ventilated hood (Field Metabolic System, Sable Systems International, Las Vegas, NV, USA). The rates of VO_2_ and VCO_2_ production were calculated using equations previously described and adapted for application with a ventilated hood [21]. This approach allowed for a constant measurement with subsequent correction for the dilution effect of water vapour pressure on VO_2_ and VCO_2_. A background baselining technique was then applied to correct for analyser drift and changes in environmental conditions. Substrate utilisation and its caloric equivalents were calculated as described previously [22].

### 2.4. Dietary 4-Day Intake Records

Dietary intake analysis, reported over the observation period, was selected to characterise the athletes’ macro- and micronutrient intake over a 4-non-consecutive-day period within the week. The intake assessment included a day of rest, a day of single practice in the afternoon, and two days of double practice in the morning and afternoon. Athletes received detailed instructions on how to record all food, fluid and dietary supplement intakes. The athletes were asked to record the day, time, type of food and amounts. Kitchen food scales were provided to facilitate the recording process. If the athletes ate out, they were instructed to provide the name of the restaurant, food, and fluid orders with size if applicable to enable cross-checking. Athletes were free to use dietary supplements, and details of these were recorded. The principal investigator (ES) reviewed all dietary records and analysis reports for consistency (ESHA The Food Processor; version 11.6.2 and Canadian Nutrient File database where possible).

### 2.5. Dual-Energy X-ray Absorptiometry Scans

The DXA scanner (Lunar Prodigy, GE Healthcare, Madison, WI, USA) was calibrated each day before measurement as per manufacturer guidelines. All scans were conducted by the same operator (ES) who was certified through the International Society of Bone Densitometry. The analysis was performed using GE Encore version 17.0 software (GE, Madison, WI, USA). The thickness mode was determined via the automated feature in the software.

Athletes were scanned after an overnight fast (≥8 h postprandial) and rest state, following a low–moderate training volume day. Athletes wore minimal clothing without metal artifacts. Athletes were positioned as previously described [23]. Participants too tall to fit within the defined scanning area undertook two scans to include the head and the remaining whole body from the inferior mandibular edge while the head was in the Frankfurt plane. All regions of interest were confirmed by the operator (ES) before inclusion for statistical analysis. Whole body fat-free mass, fat mass and % body fat were derived from the summation of the two scans. Fat-free mass and fat mass indexes were calculated by dividing fat-free mass and fat mass (in kilograms) by stature (meters) squared [24].

### 2.6. Surface Anthropometry

Anthropometric measurements of body mass, standing height, and the sums of 8 skinfolds, 3 girths, and 2 breadths were landmarked and measured by a level III International Society for the Advancement of Kianthropometry (ISAK) anthropometrist (ES). All measurements were made as previously described [25]. 

Standing height was measured using a stadiometer (Rosscraft, Surrey, BC, Canada) with a precision of ±1.0 mm. Skinfolds were assessed using Harpenden calipers (Baty International, Burgess Hill, England). Girth measurements were undertaken using a flexible steel tape (Rosscraft, Surrey, BC, Canada). The bi-epicondylar breadths were measured with a Campbell 10 small sliding bone caliper (Rosscraft, Surrey, BC, Canada). Body mass was assessed in minimal clothing on a calibrated digital scale with a precision of ±0.1 kg (BWB-800S Tanita, Arlington Heights, IL, USA). 

The calculations of lean mass index and anthropometric somatotype were performed as previously described [26,27].

### 2.7. Blood Samples

All athletes provided blood samples after a minimum 12 h overnight fast, which was analysed for complete blood count, glucose, c-reactive protein, ferritin, 25-hydroxyvitamin D and vitamin B12. All blood samples were assessed by a laboratory accredited with the Ontario Ministry of Health and Long-Term Care (Lifelabs Medical Laboratories, Ottawa, ON, Canada).

### 2.8. Victorian Institute of Sport Assessment Questionnaire—Patellar Tendon (VISA-P)

Knee health was characterised with the VISA-P. It was designed to identify clinical severity in relation to patellar tendinitis [28]. The maximum score for an asymptomatic individual is 100 points. The estimated minimal clinical important difference among athletes with chronic patellar tendinitis is a difference of 13 points [29]. All athletes completed the questionnaire 1 day prior to starting the 4-day dietary intake record.

### 2.9. Three-Factor Eating Questionnaire R-18 (TFEQ-R18)

The TFEQ-R18 refers to current dietary practice and measures 3 different aspects of eating behaviour: restrained eating (conscious restriction of food intake to control body weight or to promote weight loss), uncontrolled eating (tendency to eat more than usual due to a loss of control over intake accompanied by subjective feelings of hunger), and emotional eating (inability to resist emotional cues) [11]. The questionnaire consists of 18 items on a 4-point response scale and items are scored, summated and transformed into a 0–100 scale for each behaviour as previously described [11]. Higher scores in the respective scales indicate greater cognitive restraint, lack of control, or emotional eating. For the purpose of this investigation, emotional and cognitive restraint eating behaviours were included in our analysis. The median value was used to categorise athletes into low or high emotional and cognitive restraint eating subgroups. All athletes completed the questionnaire 1 day prior to starting the 4-day dietary intake record.

### 2.10. Statistics

Statistical analyses were carried out using SPSS (SPSS v23.0; IBM Inc., Chicago, IL, USA). Descriptive statistics are presented as mean (µ) ± standard deviation (SD). The mean intakes of protein and carbohydrate were compared to sport nutrition guidelines and mean micronutrient intakes were compared to reference intake values for a healthy general population [4,14,15,16].

Data were assessed via Shapiro–Wilk for normality and non-parametric tests were used with non-normally distributed data. Pearson’s correlation coefficient (normally distributed data) or Spearman’s correlation coefficient (non-normally distributed data) were used to identify relationships between VISA-P, body composition, somatotype and eating behaviours. Non-correction, and Bonferroni correction for multiple comparisons, were applied to define significance. 

Differences in mean VISA-P, body composition, somatotype, and nutrient intake between high and low emotional and cognitive restraint eating behaviour groups were investigated with either independent *t*-test (normally distributed data) or Mann–Whitney U test (non-normally distributed data) with significance set to *p* < 0.05. 

Differences between high and low emotional and cognitive restraint eating behaviour groups were expressed as the Cohen’s effect size with threshold values of <0.2 (trivial), 0.2–0.5 (small), 0.5–0.8 (moderate) and >0.8 (large) [30].

## 3. Results

Twenty-two athletes from a men’s national indoor volleyball program were recruited for the study. Due to time restraints, one athlete was unable to provide limb girths and bone breadths, leaving twenty-one participants for the assessment of somatotype. After a preliminary review of the dietary food intake journals, four athletes were removed from the final dietary intake analysis because of incomplete records. 

The VISA-P, anthropometric, body composition, and eating behaviour values are presented in Table 1. The mean score for VISA-P was consistent with high-risk groups for patellar tendinopathy. The mean physique morphology was ectomorphic mesomorph (2.0 ± 0.5–4.7 ± 0.7–3.3 ± 0.7) with a fat-free mass index (FFMI) and fat mass index (FMI) of 19.9 ± 1.1 and 4.1 ± 0.6 kg/m^2^, respectively. The primary eating behaviour of the group was cognitive restraint.

Macronutrient and micronutrient intake are presented in Table 2 and Table 3, respectively. Most players (83%) under-consumed carbohydrates and a notable subset (33%) under-consumed protein compared to sport nutrition reference guidelines. Additionally, most players (61%) under-consumed fiber, and a meaningful range of athletes (22–44%) had a mean intake of water, vitamin A, vitamin B12, vitamin D, thiamin, folate, vitamin E, calcium, magnesium and zinc below DRI values (Table 3). A subset of players were consuming dietary supplements that were part of the national team’s supplement provision program, such as vitamin D, whey or pea protein isolates and protein:carbohydrate:electrolyte recovery products, in addition to a smaller subset of players integrating self-supplied products such as protein bars, omega 3 or creatine monohydrate supplements (data not shown). Bloodwork identified 14% of athletes with vitamin D insufficiency (Table 4). However, the Goldberg cut-off assessment indicated an overall bias of under-reporting of energy intake in the study sample, with 21% (4 athletes) classified as under-reporters, 79% (14 athletes) as plausible reporters and no athlete as over-reporters.

A significant moderate negative correlation was observed between VISA-P and emotional eating behaviour (Table 5). The correlation assessment also showed a significant moderate positive association between cognitive restraint eating and BMI as well as FFMI, but revealed a significant moderate negative correlation with the ectomorph rating.

There was a moderate effect size between the VISA-P, mesomophic and ectomorphic ratings, and the relative protein intake between the low and high emotional eating behaviour groups. However, the differences were not significant (Table 6). Conversely, significant differences in mesomorphic and ectomorphic ratings between the low and high cognitive restraint groups were observed. Although there was a moderate effect size in the endomorphic rating, FFMI and relative fat, the differences were not significant. The overall relative energy and carbohydrate intake values were below sport nutrition guidelines in all eating behaviour groups.

## 4. Discussion

The primary finding of this investigation is that many elite indoor male volleyball players appear to under-consume energy compared to recommendations for sport [15]. This is similar to previous work involving elite-level male and female indoor volleyball players [7,8,9,10]. The deficit in energy intake may primarily be a consequence of not meeting daily carbohydrate needs for exercise [14]. The restriction of amount of and/or diversification of carbohydrate-rich foods in the overall daily diet may explain the trend of the inadequate intake of dietary fiber, thiamin, B12 vitamins, folate, vitamin A, vitamin E, vitamin D, calcium, magnesium, and zinc [31]. However, it is important to acknowledge that a 4-day dietary intake record is insufficient to accurately estimate the group’s micronutrient intake, and therefore, no definitive conclusion of risk of inadequacy is possible [32]. Although dietary intake is mediated by multiple factors, cognitive restraint eating behaviour was observed. Given the observed relationship with degree of heaviness, this behaviour may be associated with weight control in enhancing performance previously reported in other athletes [12,33]. This finding was unexpected since the group had an ectomorphic–mesomorph (lean and muscular) rating, frequently referred to as a desirable trait for elite athletes, and cases of overweight were not related to excessive body fat [33]. Nevertheless, the presence of an inadequate daily energy intake during periods of intensive training may increase the risk of undesirable health and performance outcomes from LEA, leading to relative energy deficiency in sports (RED-S) [5]. Therefore, the presence of this syndrome and its consequences for health and performance should be investigated in this group. In addition to identifying a relationship between physique and cognitive restraint eating behaviour, an association was recognised between emotional eating behaviour and knee injury. 

The undesirable consequences of patellar tendinitis were associated with emotional eating behaviour. The observed VISA-P score was consistent with high-risk groups for patellar tendinopathy. Although the difference in the VISA-P score between the high and low emotional eating behaviour groups was not statistically significant, it was borderline clinically important [29]. Emotional eating tended to increase in more symptomatic athletes. This parallels the findings in the general population, in that people in greater pain were more likely to experience emotional eating [19]. Emotional eating was associated with over-eating energy (disproportionally through sugary foods) and with struggles with obesity [19]. However, significant differences in energy intake and degree of heaviness were not observed between the emotional eating behaviour groups in this study. Perhaps the severity of the current condition was an insufficient stressor—a key mediator in the chronic pain–emotional eating behaviour model [34]. For instance, our athletes engaged in sport-specific training, were supported by experts in physical and medical sport therapies, travelled, and competed in important international events of the season. Therefore, the dissimilarities in physical disability and/or consequences to quality of life may explain the differences in dietary intake response. Alternatively, there could be a reporting bias for food choices, given that an increase in energy intake is disproportionally related to consumption of sugary foods [34]. Regardless, the lack of association between the chronic pain–emotional eating model and physique characteristics suggests that the risk of excessive energy intake may not translate to these athletes who are training and competing with injuries. Despite the nuances in eating behaviours, an important finding was that both emotional and cognitive restraint eating behaviour groups in this investigation did not report sufficient energy and carbohydrate intakes to support training demands.

Volleyball involves intermittent and frequent explosive movements [2]. The long and high-intensity bouts of exercise require athletes to have well-developed oxidative capabilities, as well as creatine phosphate and glycolytic energy systems [35]. Therefore, an athlete struggling with carbohydrate insufficiency may be at risk of reduced training quality and poor recovery, preventing them from effectively managing short-term performance readiness and long-term sport development objectives [14,36]. Additionally, in the absence of sufficient energy availability, the risk of missed training due to physical injuries/illnesses and impaired training response may be greater [5]. Interestingly, it may be worth exploring the relationship between LEA and patellar tendinitis given the undesirable consequences of impaired collagen synthesis in bone tissue [37]. Identifying a notable relationship could inform dietary approaches to enhance the management of knee health in this population. Overall, the integration of expert nutrition support to develop suitable and practical nutrition solutions for players would be beneficial, especially during intensive and critical phases of the competitive season. 

It is important to examine the limitations of our investigation so as to inform the interpretation of results. Several decisions related to study design impacted the assessment of dietary intake. First, a 4-day intake record was applied to minimise player distraction while preparing for Olympic qualification. There is a risk of increased variance in the estimates of nutrient intake in the 4-day compared to the 7-day observation method [38]. This may influence the accuracy of our estimates of energy and macronutrient intake, and limit our ability to assess differences between low and high emotional and cognitive restraint eating behaviour groups. Second, the common problem of under-reporting nutrient intake in athletes may impact our results [39]. This issue was observed given that four athletes were unable to complete their dietary intake journals, and an overall bias of under-reporting was revealed in our sample. However, the validation assessment of energy intake is unable to discriminate between under-reporting and dieting. Our study cohort demonstrated a restraint eating behaviour profile, which was associated with body mass index and ectomorph (linearity) rating, and therefore, some athletes may have intentionally reduced their energy intake, or maintained a low one, for control over body weight and/or physique morphology [40]. The association between physique and eating behaviour also suggests an increased risk of restrained eating in heavier athletes, despite levels of fat mass. Finally, significant associations with eating behaviours were only observed when pairwise comparisons were not controlled for familywise error rate. Overall, we consider this assessment preliminary, and encourage replication. These limitations offer a reminder of the difficulties associated with the assessment and interpretation of dietary intake data.

## 5. Conclusions

In conclusion, elite male indoor volleyball players are at risk of consuming inadequate energy and carbohydrate for their sport. This may impair players’ ability to adapt to training and to benefit from recovery during intensive training periods over a competitive season. Moreover, athletes with restrictive eating behaviour may be at additional risk of injuries and impaired performance associated with LEA and/or macronutrient and/or micronutrient intake insufficiency. Future work should explore the presence of LEA in elite male indoor volleyball players and describe its consequences for health and performance.

## Figures and Tables

**Table 1:** Player metabolism, knee health, physique traits and eating behaviours (*n* = 22).

Category	Variables	µ ± SD
Demographic	Age (years)	25.8 ± 3.2
Metabolism	Measured Resting Metabolic Rate (kcal)	2419 ± 393
Knee Health	VISA-P Score	80.8 ± 14.6
Physique Traits	Standing Height (cm)	197.2 ± 8.1
	Body Mass (kg)	93.3 ± 8.8
	∑8 Skinfolds (mm)	74.2 ± 15.4
	Lean mass index (mm∙kg^−0.14^)	52.6 ± 5.2
	Fat free mass (kg)	77.5 ± 7.7
	Fat mass (kg)	15.9 ± 2.6
	Body Fat (%)	17.0 ± 2.2
	Fat free mass Index (kg/m^2^)	19.9 ± 1.1
	Fat mass Index (kg/m^2^)	4.1 ± 0.6
	Body Mass Index (kg/m^2^)	24.0 ± 1.2
	Endomorphy ^a^	2.0 ± 0.5
	Mesomorphy ^a^	4.7 ± 0.7
	Ectomorphy ^a^	3.3 ± 0.7
Eating Behaviours	Emotional Eating	14.4 ± 14.6
	Cognitive Restraint Eating	30.6 ± 17.1

µ = mean; SD = standard deviation; ^a^ *n* = 21, 1 athlete removed due to missing data. VISA-P = Victorian Institute of Sport Assessment questionnaire—patellar tendon.

**Table 2:** Energy and macronutrient intake as well as proportion of players at risk of not meeting dietary intake guidelines (*n* = 18).

Nutrients	µ ± SD	Min	Max	Below DRI, % (*n*)	DRI or Sport Nutrition Reference
Energy (kcal/day)	3034 ± 1345	1965	4835	-	-
Energy (kcal/kg/day)	33 ± 15	21	50	78 (14)	40–70 ^e^
Carbohydrate (g/day)	325 ± 105	179	560	-	-
Carbohydrate (g/kg/day)	3.5 ± 1.3	1.9	6.1	83 (15)	5–7 ^b^
Carbohydrate (% total energy)	42 ± 8	28	56	72 (13)	45–65 ^d^
Fiber (g/day)	40 ± 17	15	81	61 (11)	38 ^a^
Protein (g/day)	161 ± 34	112	240	-	-
Protein (g/kg/day)	1.7 ± 0.4	1.2	2.6	33 (6)	1.6 ^c^
Protein (% total energy)	22 ± 4	16	29	0 (0)	10–35 ^d^
Fat (g/day)	119 ± 37	68	238	-	-
Fat (g/kg/day)	1.2 ± 0.3	0.8	2.2	-	-
Fat (% total energy)	35 ± 7	22	44	0 (0)	20–35 ^d^
Saturated Fat (g/day)	33 ± 16	17	51	-	-
Saturated Fat (% total energy)	10 ± 5	5	15	-	-
Omega 3 (g/day)	2 ± 3	1	11	-	-
Omega 3 (% total energy)	1 ± 1	0	2	-	-

µ = mean; SD = standard deviation, DRI = dietary reference intake; ^a^ recommended daily allowances, Otten et al., 2006. ^b^ Burke et al., 2011. ^c^ Morton et al., 2018. ^d^ Acceptable macronutrient distribution ranges, Otten et al., 2006. ^e^ Kerksick et al., 2018.

**Table 3:** Micronutrient intake of players compared to DRI values (*n* = 18).

Nutrients	Intake, µ ± SD	Intake, % DRI, µ ± SD	Below DRI, % (*n*)	Below EAR, % (*n*)	DRI ^a^
Thiamin (mg/day)	1.3 ± 0.4	111 ± 35	44 (8)	28 (5)	1.2
Riboflavin (mg/day)	2.1 ± 0.7	160 ± 54	11 (2)	6 (1)	1.3
Niacin (mg/day)	21 ± 6	131 ± 40	28 (5)	11 (2)	16
Vitamin B6 (mg/day)	2.3 ± 0.8	175 ± 59	5 (1)	0 (0)	1.3
Vitamin B12 (mcg/day)	4.6 ± 3.7	192 ± 153	28 (5)	28 (5)	2.4
Folate (mcg/day)	438 ± 150	110 ± 37	56 (10)	22 (4)	400
Vitamin A (mcg/day)	813 ± 341	90 ± 38	67 (12)	22 (4)	900
Vitamin D (IU/day)	296 ± 264	148 ± 132	39 (7)	-	200 ^b^
Vitamin C (mg/day)	241 ± 334	268 ± 371	11 (2)	6 (1)	90
Vitamin E (mg/day)	14 ± 9	90 ± 62	56 (10)	50 (9)	15
Calcium (mg/day)	1256 ± 493	126 ± 49	22 (4)	-	1000 ^b^
Iron (mg/day)	24 ± 9	297 ± 108	-	0 (0)	8
Magnesium (mg/day)	413 ± 217	103 ± 54	56 (10)	44 (8)	400
Zinc (mg/day)	11 ± 5	100 ± 44	61 (11)	44 (8)	11
Water (g/day)	4433 ± 1098	120 ± 30	28 (5)	-	3700
Water (g/kg/day)	48.4 ± 13.3	-	-	-	-

µ = mean; SD = standard deviation; DRI = dietary reference intake; EAR = estimated average requirement; ^a^ recommended daily allowance, male 19–30 years old; ^b^ adequate intake, male 19–30 years old.

**Table 4:** Hematological parameters for assessment of nutrient deficiencies (*n* = 22).

Parameters	µ ± SD	Below Normal Range, % (*n*)	Reference Value ^a^	Clinically Significant Values
Red blood cell count (×10^12^/L)	4.86 ± 0.56	9 (2)	4.50–6.00	Athlete 1 = 4.42 Athlete 2 = 4.42
Hemoglobin (g/L)	150.2 ± 8.9	9 (2)	135–175	Athlete 1 = 128
Hematocrit (L/L)	0.440 ± 0.050	5 (1)	0.400–0.500	Athlete 1 = 0.384
MCV (fL)	89.4 ± 3.0	0 (0)	80–100	
MCH (pg)	30.3 ± 1.4	5 (1)	27.5–33.0	Athlete 3 = 27.3
Fasting blood glucose (mmol/L)	4.8 ± 0.4	0 (0)	3.6–6.0	
Vitamin B12 (pmol/L)	317.5 ± 113.4	0 (0)	138–652	
Ferritin (µg/L)	109.0 ± 34.7	0 (0)	22–275	
c-Reactive Protein (mg/L)	1.6 ± 4.1	5 (1)	<5.0	Athlete 4 = 19.3
25-hydroxyvitamin D (nmol/L)	96.7 ± 27.9	14 (3)	75–250	Athlete 4 = 64 Athlete 5 = 69 Athlete 6 = 72

µ = mean; SD = standard deviation; MCV = mean corpuscular volume; MCH = mean cell hemoglobin; ^a^ Lifelabs Inc., 2019.

**Table 5:** Correlation between eating behaviours, knee health, and physique traits (*n* = 22).

		1	2	3	4	5	6	7	8
**1**	Emotional Eating								
**2**	Cognitive Restraint Eating	0.35							
**3**	VISA-P Score	−0.48 *	−0.09						
**4**	Fat free mass Index (kg/m^2^)	0.25	0.37 *	−0.48 *					
**5**	Fat mass Index (kg/m^2^)	0.00	0.25	0.08	0.14				
**6**	Body Mass Index (kg/m^2^)	0.18	0.38 *	−0.37 *	**0.89**	**0.52**			
**7**	Endomorphy ^a^	0.13	0.30	0.17	0.24	**0.65**	0.48 *		
**8**	Mesomorphy ^a^	0.25	0.29	−0.12	**0.57**	0.35	**0.68**	**0.62**	
**9**	Ectomorphy ^a^	−0.23	−0.40 *	0.03	**−0.63**	−0.50 *	**−0.77**	**−0.68**	**−0.78**

**Bold** indicates significance after correction by Bonferroni method (*p* < 0.01). * Significant *p* < 0.05. ^a^ *n* = 21, 1 athlete removed due to missing data. VISA-P = Victorian Institute of Sport Assessment questionnaire—patellar tendon.

**Table 6:** Difference in physique trait, knee health and nutrient intake between low and high eating behaviours (*n* = 22).

	Emotional Eating (µ ± SD)			Cognitive Restraint Eating (µ ± SD)		
	Low	High	ES	Low	High	ES
Eating Behaviour Score	2.3 ± 10.4	26.5 ± 10.4 *	2.33	18.0 ± 7.2	43.9 ± 13.0 *	2.46
VISA-P	85.6 ± 12.5	76.0 ± 15.4	0.68	82.7 ± 12.5	79.2 ± 16.5	0.24
Fat-Free Mass Index (kg/m^2^)	19.6 ± 0.9	20.2 ± 1.2	0.57	19.4 ± 0.8	20.2 ± 1.2	0.78
Fat Mass Index (kg/m^2^)	4.1 ± 0.8	4.1 ± 0.5	0.00	3.9 ± 0.7	4.1 ± 0.6	0.31
Endomorphy ^a^	1.9 ± 0.6	2.1 ± 0.5	0.36	1.8 ± 0.4	2.2 ± 0.6	0.78
Mesomorphy ^a^	4.4 ± 0.7	4.9 ± 0.7	0.71	4.3 ± 0.7	4.9 ± 0.7 *	0.86
Ectomorphy ^a^	3.5 ± 0.6	3.1 ± 0.8	0.57	3.7 ± 0.6	3.0 ± 0.7 *	1.07
Energy (kcal/kg/d) ^b^	32.5 ± 8.9	33.4 ± 8.3	0.10	33.9 ± 8.4	32.5 ± 8.6	0.16
Protein (g/kg/d) ^b^	1.6 ± 0.3	1.8 ± 0.4	0.57	1.7 ± 0.3	1.8 ± 0.4	0.28
Carbohydrate (g/kg/d) ^b^	3.5 ± 1.1	3.5 ± 1.6	0.00	3.6 ± 1.2	3.5 ± 1.5	0.07
Fat (g/kg/d) ^b^	1.3 ± 0.4	1.2 ± 0.2	0.31	1.4 ± 0.4	1.2 ± 0.3	0.57
Fiber (g/d) ^b^	40.9 ± 18.7	38.6 ± 17.3	0.13	40.7 ± 8.2	38.9 ± 15.4	0.10

µ = mean; SD = standard deviation; ES = effect size (<0.2 (trivial), 0.2–0.5 (small), 0.5–0.8 (moderate) and >0.8 (large)); * significant difference (*p* < 0.05) between low and high eating behaviour. ^a^ *n* = 21, ^b^ *n* = 18.
